# Moving in an Environment of Induced Sensorimotor Incongruence Does Not Influence Pain Sensitivity in Healthy Volunteers: A Randomised Within-Subject Experiment

**DOI:** 10.1371/journal.pone.0093701

**Published:** 2014-04-07

**Authors:** Benedict Martin Wand, Lareina Szpak, Pamela J. George, Max K. Bulsara, Neil Edward O’Connell, G. Lorimer Moseley

**Affiliations:** 1 School of Physiotherapy, The University of Notre Dame Australia, Fremantle, Western Australia, Australia; 2 Floreat Physiotherapy, Floreat, Western Australia, Australia; 3 Institute for Health Research, The University of Notre Dame Australia, Fremantle, Western Australia, Australia; 4 Centre for Research in Rehabilitation, School of Health Sciences and Social Care, Brunel University, Uxbridge, United Kingdom; 5 The Sansom Institute for Health Research, University of South Australia, Adelaide, South Australia, Australia; University of Montreal, Canada

## Abstract

**Objectives:**

It has been proposed that in the same way that conflict between vestibular and visual inputs leads to motion sickness, conflict between motor commands and sensory information associated with these commands may contribute to some chronic pain states. Attempts to test this hypothesis by artificially inducing a state of sensorimotor incongruence and assessing self-reported pain have yielded equivocal results. To help clarify the effect sensorimotor incongruence has on pain we investigated the effect of moving in an environment of induced incongruence on pressure pain thresholds (PPT) and the pain experienced immediately on completion of PPT testing.

**Methods:**

Thirty-five healthy subjects performed synchronous and asynchronous upper-limb movements with and without mirror visual feedback in random order. We measured PPT over the elbow and the pain evoked by testing. Generalised linear mixed-models were performed for each outcome. Condition (four levels) and baseline values for each outcome were within-subject factors.

**Results:**

There was no effect of condition on PPT (p = 0.887) or pressure-evoked pain (p = 0.771). A sensitivity analysis using only the first PPT measure after each condition confirmed the result (p = 0.867).

**Discussion:**

Inducing a state of movement related sensorimotor incongruence in the upper-limb of healthy volunteers does not influence PPT, nor the pain evoked by testing. We found no evidence that sensorimotor incongruence upregulates the nociceptive system in healthy volunteers.

## Introduction

There is increasing evidence that a number of chronic pain conditions are characterised by functional and structural changes within the brain [1]–[4]. Some authors have suggested that these changes may be maladaptive and contribute, at least in part, to the maintenance of the chronic pain state [5]–[7]. A mismatch between the brain's motor control and sensory systems has been suggested as one mechanism whereby maladaptive neuroplastic changes might contribute to the experience of chronic pain [8], [9]. When a motor command is created, the central nervous system makes predictions of the sensory consequences of the movement and monitors the congruence between predicted and actual sensory feedback [10], [11]. If incongruence is detected, it is hypothesised that pain may arise to warn of an error in information processing [8]. Pain induced disruption of cortical somatosensory representation and subsequent distortion of body perception are considered possible mechanisms underpinning the production of sensorimotor incongruence in clinical populations [8], [12].

It is possible to artificially create incongruence between motor intent and the sensory feedback associated with movement using mirrors. For example, if one hand is placed in a mirror box and the other hand alongside the mirror such that its reflection appears in the space where the hidden hand should be, incongruence between motor intent, proprioception and visual feedback can be achieved by performing asynchronous bilateral wrist flexion and extension. While the intention will be to move both hands out of phase, such that while one is extending the other is flexing, visual feedback will show both hands moving in unison.

Several authors have attempted to experimentally test the sensorimotor incongruence hypothesis using this methodology. These studies suggest that visually mediated incongruence triggers altered sensation in healthy volunteers [13]–[15], fibromyalgia patients [14], symptomatic and non-symptomatic violin players [16] and patients with acute [17] or chronic [15] whiplash and there is some suggestion that these changes are more pronounced when incongruence is maximised [13], [15], [16]. However, the influence of sensorimotor incongruence on pain is harder to interpret. Some studies report no pain with incongruent movement in healthy volunteers [15], [16] and no study to date has found a positive relationship between the intensity or frequency of pain and the extent of sensorimotor incongruence [13], [14]. Furthermore, an alternative approach to inducing incongruence using tendon vibration found that vibration induced incongruence created feelings of peculiarity, foreignness and swelling, but not pain or discomfort [18].

The data to date appear to suggest that sensorimotor incongruence induces various sensory changes, however the effect on pain is less clear. One possibility is that incongruence upregulates the nociceptive/pain system but not sufficiently to evoke pain. A more sensitive method would be to load the nociceptive system by applying standardised noxious stimuli, for example using pressure pain threshold testing. Our aim was to determine if sensorimotor incongruence leads to upregulation of the nociceptive/pain system to help clarify the effect incongruence has on pain. To this end we performed a randomised cross-over experiment in which healthy subjects performed synchronous and asynchronous upper limb movements with and without mirror visual feedback. We measured pressure pain threshold (PPT), and the pain experienced on completion of PPT testing, over the elbow of the non-dominant arm immediately after each movement condition. We hypothesised that PPT would be lower, and the resultant pain would be higher, when participants performed asynchronous movements with mirror visual feedback (the condition of maximal sensorimotor incongruence) than when they performed the other three conditions.

## Materials and Methods

### Design and Ethics Statement

The study utilised a randomised, cross-over design and was approved by the Human Research Ethics Committee of The University of Notre Dame Australia (Ref # 011007F). Participants provided informed consent by signing a written consent form and all procedures conformed to the Declaration of Helsinki.

### Participants

Thirty-five healthy volunteers were recruited from staff, their families and students at the University of Notre Dame Australia. Participants were eligible if they were right handed (determined by hand used for writing), between 18 – 60 years of age, fluent in written and spoken English and able to provide informed consent. Subjects were excluded if they had any ongoing medical complaint, had experienced any musculoskeletal pain in the past six-months, had experienced any episode of upper limb pain that had restricted work or leisure or required a visit to a health care professional in the last two years, had significant visual impairment, had previous surgery involving either upper limb, had any significant asymmetrical visual disfigurement on their upper limbs (including tattoos) or were currently taking any psychoactive medication. The participants were naïve to the sensorimotor incongruence theory and blinded to the hypotheses of the study.

### Procedure

Before testing, basic demographic data were collected, consent was obtained and each participant was assigned a research number. A counter-balanced random number sequence was computer-generated by an individual not involved with the study and each number was placed in consecutively numbered, sealed, opaque envelopes. After completion of the baseline assessment an independent researcher opened the envelope that corresponded to the participant's research number and the number dictated the order in which that participant undertook the conditions.

For all movement conditions, participants were seated and the entire upper limb exposed (participants wore a sleeveless t-shirt and removed all jewellery). A height adjustable table was positioned on the participant's left hand side and adjusted so that the subject could position their left arm on the table with the shoulder at 90° abduction, the elbow at 90° flexion and the forearm pronated. Each movement condition lasted 40 seconds. A metronome set at 1.3 Hz was used to standardise the speed of movement and the number of movement repetitions. Movement excursion was monitored in real time and feedback given to ensure range of motion remained the same within and between each condition. Immediately on completion of each condition the participant was instructed to position their left arm on the table for PPT testing. The assessor who performed the PPT testing was in the room behind a screen next to the table wearing sound cancelling headphones. On completion of each condition the assessor was tapped on the shoulder, they then removed their headphones and immediately completed the first PPT test.

**Figure 1:**
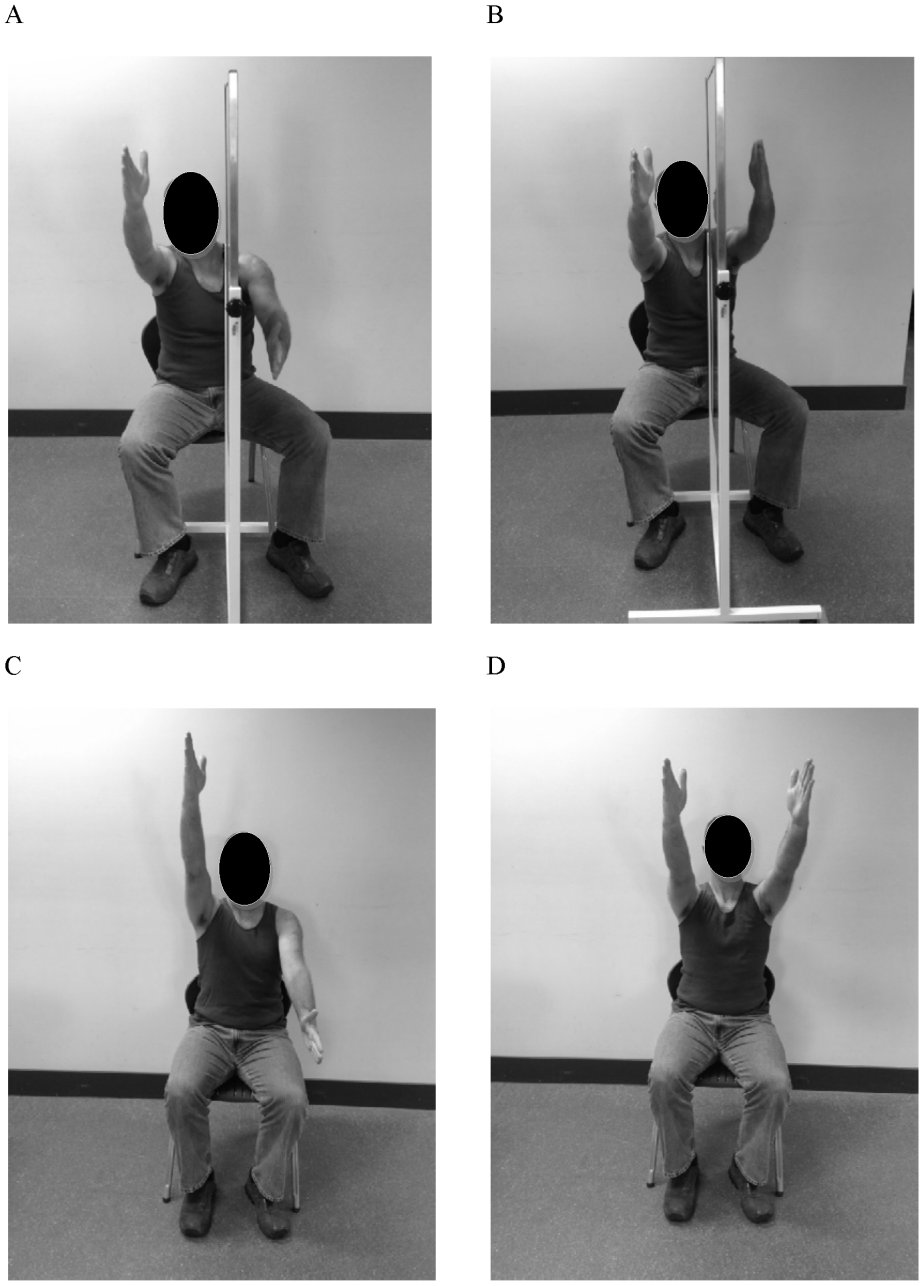
Experimental set up. **A**: asynchronous movements with mirror. **B**: synchronous movements with mirror. **C**: asynchronous movements without mirror. **D**: synchronous movements without mirror.

There were two mirror visualisation conditions and two normal visualisation conditions. For the mirror visualisation conditions, a large mobile mirror was placed in line with the participant's parasagittal axis with the reflective surface facing the subject's right side. The room was set up symmetrically, with plain white walls on either side to enhance the illusion of ownership over the reflected arm. The arms were placed either side of the mirror and the participant was instructed to attend to the reflection of the right arm in the mirror. The left arm was therefore hidden from view and the reflected right arm appeared to be in the space that the left arm would normally occupy (see Figure1). For the synchronous movement condition, both upper limbs were positioned with the thumbs facing up and simultaneous repeated flexion and extension at the shoulder were performed in time to the metronome. For the asynchronous condition the participant initially performed ten repetitions of simultaneous repeated shoulder flexion and extension and then, on instruction from a research assistant, switched to alternate flexion and extension for the remainder of the forty seconds. We adopted this strategy because in pilot testing we found that the illusion of ownership of the reflected image was reduced when the initial movements were asynchronous. Throughout the task participants were instructed to attend to the mirror and follow the reflected image of their arm as it moved. At the completion of each experimental condition, the mirror was moved to a standardised position in the room so as to ensure that the researcher performing the PPT testing was blinded to condition.

The normal visualisation conditions were identical except that no mirror was used and the participant attended to their *actual* left arm during the performance of either synchronous or asynchronous upper limb movements. The mirror was left in the standardised position throughout testing. A fifteen-minute washout period separated conditions.

### Outcome Measures

#### Pressure Pain Threshold

We defined PPT as the minimal amount of force where a sense of pressure first changes to pain [19]. PPT was measured using a hand-held pressure algometer (Wagner instruments, Greenwich, CT, USA) consisting of a 1 cm^2^ round rubber disk attached to a pressure gauge, which displayed force digitally in increments of 0.1 N/cm^2^. The testing protocol outlined by Chesterton et al [20] was used, including an initial training period to ensure standardisation of rate of force development. This protocol has demonstrated excellent reliability (ICC 0.91) [20]. Weekly calibration of the algometer, and of the tester's rate of force development, was undertaken. All testing was carried out by the same assessor who was blind to movement condition.

Prior to formal testing, the procedure and testing approach were explained. Fifteen test runs on the leg and one test run over the lateral epicondyle of the right arm allowed familiarisation with the procedure. For formal data collection, a point on the left forearm, 2 cm distally and medially to the lateral epicondyle of the humerus in the belly of the wrist extensor muscles was localised by palpation and marked with a felt-tipped pen [19]. This mark established the site for all testing. We chose to test over a muscle unlikely to be active during the movement performed to avoid contamination from movement induced muscle soreness. The tester applied force at a standardised rate until the participants' first perception of pain; at which point pressure was released. The algometer reading was recorded by another researcher to ensure both the participant and tester were blind to the PPT values. On each occasion of testing three readings were taken from the marked area on the left forearm and the mean of these three values was used for statistical analysis [20]. A fifteen second rest period was given between each of the three readings [20]. A baseline threshold measurement was taken fifteen minutes before any movement commenced and subsequent measures were taken immediately on completion of each of the four movement conditions. To assess integrity of the blinding, at the completion of all assessments the tester was asked to indicate which condition they thought was the experimental condition.

#### Pain Intensity

As a secondary measurement of sensitivity, and to maybe capture evidence of enhanced sensitisation, we also asked subjects to rate pain intensity specifically at the site of testing on completion of the PPT testing – care was taken to explain that we were interested in recording the pain intensity felt at the testing site now, not the pain intensity associated with the PPT testing. Pain intensity was measured using a numerical rating scale (NRS) anchored 0 =  ‘no pain’ and 10 =  ‘pain as bad as you can imagine’. Pain intensity was measured after baseline PPT testing and on completion of each of the four movement conditions.

### Sample Size

A power calculation for a cross-over design was performed for the primary outcome measure of PPT over the lateral epicondyle. A two-sided t-test achieves 80% power to infer that the mean difference is not 0 when the total sample size for a cross-over design is 35, the actual mean difference is 2.5 N/cm^2^, the standard deviation of the differences is 5 [19], and the significance level is 0.05, indicating that a total of 35 patients were required for our study.

### Data Analysis

#### Participant Characteristics and Methodological Checks

Descriptive statistics were used to report patient demographic information. The repeatability of PPT testing was assessed by calculating the intra-class correlation coefficients (ICC) for each of the three values taken at the baseline assessment. A two-way random effects model was used to determine the ICC value. To assess for integrity of blinding the comparison of proportions was done using the Chi-Squared Test with Fishers exact p-value. For each dependant variable, the order effect of condition was checked using a generalised linear mixed model.

#### The Effect of Movement Condition on PPT

Due to the correlated nature of the data collected, a regression based generalised linear mixed model was used to explore the relationship between the dependent variable (PPT) and condition, which were treated as independent fixed-effects variables (within-subject factors). The baseline measurement of PPT was entered as a covariate in this analysis. The null hypothesis was that the difference in mean PPT for each condition was not significant. A Bonferroni correction was used for post hoc comparisons.

We also undertook a sensitivity analysis of our primary hypothesis by repeating the generalised linear mixed model using only the first PPT measurement. This measure was recorded immediately after movement and was therefore most likely to detect changes induced by the experimental conditions. In this analysis the baseline measurement of the first PPT value was used as a covariate.

#### The Effect of Movement Condition on Pain Intensity

To determine if moving in an environment of induced sensory-motor incongruence influenced the intensity of local pain that results from PPT testing, the pain intensity scores obtained post PPT testing of each subject were analysed using a generalised linear mixed model similar to that described above. Baseline pain intensity scores were used as a covariate in this analysis.

## Results

### Participant Characteristics

The average age of participants was 27 years (SD 11), 31% were male. The average height was 173 cm (SD 9) and average weight 73 kg (SD 15).

### Methodological Checks

All 35 participants completed all phases of the study and there were no missing data. The repeatability (ICC) of PPT readings for the elbow determined for the three repeated measures collected at baseline was 0.95 (95% CI: 0.93-0.96). Twelve out of 35 times (34%), the observer correctly identified the maximal incongruence condition as the experimental condition. This was not statistically significant (p = 0.219) when compared to the expected proportion of 25%. There was no order effect of movement condition on average PPT (p = 0.307), the first PPT measurement (p = 0.587) or pain intensity (p = 0.151).

### Baseline Assessment

The mean (SD) baseline values of the three outcome measures were: PPT = 34.6 (27.56); first PPT = 36.46 (27.85) and pain intensity  = 0.94 (1.14).

### The Effect of Movement Condition on PPT

Table 1 shows the mean and standard deviation (SD) of the PPT measure for all 35 subjects after each condition. The generalised linear mixed model analysis showed no statistically significant difference for each bivariate comparison of condition (p = 0.887), indicating that there was no difference in PPT between the different movement conditions. The mean difference (and 95% CI) in PPT scores between the experimental condition and each of the control conditions is given in Table 2.

**Table 1:** Mean (SD) of each outcome measure across each condition (after adjusting for baseline values).

Outcome	Condition				Test for difference: p-value
	Asynchronous Mirror (n = 35)	Synchronous Mirror (n = 35)	Asynchronous No Mirror (n = 35)	Synchronous No Mirror (n = 35)	
Average PPT (SD) N/cm^2^	33.2 (28.60)	31.5 (25.18)	32.6 (29.85)	31.2 (25.06)	0.887
First PPT measurement (SD) N/cm^2^	34.8 (29.80)	32.5 (24.12)	34.1 (30.97)	32.8 (25.66)	0.867
Pain Intensity after PPT testing (SD) NRS/10	1.4 (1.36)	1.2 (1.36)	1.5 (1.52)	1.3 (1.28)	0.771

PPT  =  Pressure pain threshold

NRS  =  Numerical rating scale

**Table 2:** Mean differences and 95%CI for each outcome measure.

Outcome	Asynchronous Mirror – Asynchronous No Mirror	Asynchronous Mirror – Synchronous No Mirror	Asynchronous Mirror – Synchronous Mirror
Average PPT (95% CI) N/cm^2^	0.64 (−5.01 – 6.30) p = 0.822	2.00 (−3.64 – 7.63) p = 0.484	1.70 (−3.94 – 7.33) p = 0.552
First PPT measurement (95% CI) N/cm^2^	0.70 (−5.39 – 6.80) p = 0.820	1.95 (−4.13 – 8.02) p = 0.527	2.28 (−3.80 – 8.36) p = 0.459
Pain Intensity after PPT testing (95% CI) NRS/10	−0.15 (−0.80 – 0.50) p = 0.646	0.10 −0.55 – 0.75) p = 0.756	0.18 (−0.47 – 0.83) p = 0.593

Effect sizes are given for the experimental condition (asynchronous mirror) in comparison to each control condition.

The mean and SD of the first PPT, recorded immediately after each condition, can also be found in Table 1. The results of the sensitivity analysis using only the first PPT test yielded the same results. There was no statistically significant difference found for the bivariate comparisons of condition (p = 0.867), demonstrating that there was no difference in PPT taken immediately post movement between the different movement conditions. The mean difference (and 95% CI) in first PPT scores between the experimental condition and each of the control conditions is given in Table 2.

### The Effect of Movement Condition on Pain Intensity

Table 1 shows the mean and SD for pain intensity associated with PPT testing of the 35 subjects after each condition. The analysis showed no statistically significant difference for the bivariate comparison of condition (p = 0.771), signifying no difference in pain intensity across the four movement conditions. The mean difference (and 95% CI) in pain intensity scores between the experimental condition and each of the control conditions is given in Table 2.

## Discussion

The aim of this study was to investigate whether moving in an environment of induced sensorimotor incongruence leads to upregulation of the nociceptive system. We artificially created incongruence between motor intent and the sensory feedback associated with movement, by asking participants to perform repeated asynchronous arm movements with mirror visual feedback and then measured the pressure pain threshold over the elbow and the pain intensity experienced at the completion of PPT testing immediately after this movement task. We also assessed participants immediately after asynchronous movement without a mirror, synchronous movement without a mirror and synchronous movement with a mirror, to control for the confounding influences of arm movement, asynchronous movement and visualisation of the reflection of the arm. Contrary to our hypothesis, PPT was not reduced during the incongruent condition, and in fact was near identical after each of the four movement stages. A sensitivity analysis in which we used only the first PPT test (recorded immediately on movement cessation) returned the same result. Furthermore, the self reported pain intensity felt after PPT testing was the same across all four conditions. Using a highly reliable and sensitive measure of pain sensitivity we found no evidence of upregulation of the nociceptive/pain system when healthy subjects move in an environment of induced sensorimotor incongruence.

To date, the direct experimental support for the contribution of sensorimotor incongruence to the experience of pain is inconsistent. In early studies most healthy participants did not report any pain [13], [14], and for those who did there was no clear relationship between increasing incongruence and the report of painful symptoms. For example, when healthy volunteers performed arm movements either side of a mirror, discomfort appeared similar whether the arms were moved asynchronously (maximal incongruence) or synchronously (minimal incongruence) [13], [14]. It is possible that methodological issues contributed to the report of pain in these studies, particularly the influence of suggestion over participant's responses, as there was no blinding of assessors [21]. In more recent and methodologically rigorous studies artificially inducing sensorimotor incongruence have produced numerous sensory changes but minimal reported pain in healthy pain free controls [15], [16], [18], [22]. When visual feedback from the moving limb is manipulated in clinical populations, it seems that reports of pain are more common than in healthy participants [14], [15], [17]. However, as with the healthy population, there appears to be no difference in reported pain between conditions of minimal and maximal incongruence [14]. Our findings are in agreement with the more recent and methodologically robust studies which have found minimal reports of pain when sensorimotor incongruence is induced in healthy volunteers [15], [16], [18], [22]. The current results also seem consistent with the recent investigation, by two independent research groups, using a blinded randomised design, which showed that the rubber hand illusion, which introduces a mismatch between proprioceptive and visual feedback, does not modulate experimentally induced thermal pain in healthy volunteers [23].

Although the evidence is building that sensorimotor incongruence does not produce pain in a healthy nervous system, it remains possible that it does produce pain in a pathological nervous system, such as might be found in someone with chronic pain [2], [24], [25]. This position would be consistent with the contrasting effects of magnifying the visual image of a painful body part, which has an analgesic effect in healthy volunteers experiencing experimentally induced pain [26] but increases pain in patients with CRPS who are moving their painful limb [27]. Alternatively, perhaps the mounting evidence against the sensorimotor incongruence idea in healthy volunteers also applies to patients. The most robust studies in whiplash patients support this position [15], [17] and in participants with fibromyalgia, a condition characterized by heightened pain sensitivity, neither the report of pain nor any other sensory symptoms appear to be greater in the condition of maximal sensorimotor incongruence [14]. If this is the case, it appears important to revisit the proposed mechanism of treatments for chronic pain such as mirror therapy.

Mirror visual feedback therapy seems to reduce pain in some chronic limb pain states [28], [29] (see [30] for review and [31] for meta-analysis) and preliminary data suggest that visualisation of the back during movement reduces pain in low back pain patients [32]. These effects have been interpreted to possibly reflect improved sensory acuity of the affected area and re-establishment of the normal pain-free relationship between sensory feedback and motor intention [33]. Both tactile [34] and proprioceptive [35] acuity are enhanced with visualisation of the area, and it seems plausible that these and other perceptual impairments could be rectified by mirror visual feedback and thus help normalise the relationship between actual and intended movement. Perhaps however, the effect is unrelated to sensorimotor incongruence. Other mechanisms have been proposed – it is possible that the illusion created by the mirror of a painful limb that now looks normal may result in the brain rejecting nociceptive input as spurious because there is no other evidence that the limb is in danger [36] – that is ‘all is as it should be’ [24]. Perhaps the appearance of a normal limb in place of the painful one reduces the anxiety and fear of movement and the threat value associated with use of the painful area [33]. It is also possible that cross-modal inhibition, or an increased sense of bodily ownership and control, imparts the effect [37]. Finally, perhaps mirrors just offer a very engaging and almost ‘magical’ distraction.

Our findings suggest that a mismatch between movement intent and feedback might not contribute to clinical pain states; however it is important that the results of the current study are interpreted with regard to the design limitations. Firstly, the perception of threat is an important component of construction of the pain experience [38]. The experimental production of sensorimotor incongruence we used probably lacked a threatening context as there is a plausible and short-lived reason for the mismatch between movement intent and feedback. The experience of an individual moving briefly in an environment of sensorimotor incongruence produced by mirrors is likely to be different from a patient moving in an environment where incongruence may be derived from cortical reorganisation and resultant altered self perception. The use of alternative strategies to create incongruence such as virtual reality technology may offer an experimental paradigm closer to the clinical experiences of pain patients and may yield different outcomes to those reported here. Additionally, time might be an important factor. The evidence seems clearer that incongruence leads to altered sensations, and these features may be the precursor to upregulation of the nociceptive system. Feelings of foreignness, peculiarity and other sensory changes may signal that the body part is not functioning normally and is in need of protection; enhanced nociceptive efficiency and pain may be later consequences of these sensory changes. Whether this can be meaningfully captured with the experimental paradigms currently in use is difficult to determine. Most previous studies employed movement times of 20 seconds [13]–[17], in order to minimise the possible influence of pain associated with muscle fatigue. We felt our testing procedure was less likely to be influenced by fatigue so increased the movement time to 40 seconds, yet we were still unable to detect any increase in sensitivity; however we cannot rule out that different results may occur with longer movement times. Also, it is only possible to accurately measure PPT when the limb is stationary. If incongruence generates a transient upregulation of nociceptive sensitivity then it is possible that by testing after the condition we missed the effect. Our sensitivity analysis suggests against this possibility but it cannot be excluded. Previous investigations have also used movement of the arm hidden behind an opaque screen as an additional control condition [13]–[17], [22]. Data suggest, and indeed the authors have argued, that movement with the arm hidden may itself offer some degree of sensorimotor discordance [14]. The mirror – non mirror contrast used in our study is likely to offer a clearer congruent - incongruent movement distinction and therefore greater potential to identify any differences in pain sensitivity had they been apparent. In addition, there is little empirical data to support the use of a 15 minute washout period. However, we detected no order effect for any outcome measure and the near identical results for all conditions suggest that adequate washout was achieved. There is also a possibility whenever no effect is detected, that the study was underpowered to detect an effect. We powered our study to detect the smallest clinically relevant effect yet none of the comparisons approached significance. It is feasible that an effect exists, but we contend that, if so, it must be very small indeed. Our study was strengthened by the inclusion of blinded assessment and a methodological check for the efficacy of this blinding. We recommend similar measures should be included in patient-targeted replications of the current work.

In conclusion, our results do not support the hypotheses that PPT would be lower, and the pain experienced immediately on completion of PPT testing would be higher, when participants performed asynchronous movements with mirror visual feedback (the condition of maximal sensorimotor incongruence) than when participants performed conditions involving less or no sensorimotor incongruence. The inability to detect any upregulation of the nociceptive system using a sensitive and reliable measure and utilising a robust and blinded design questions the role sensorimotor incongruence might have in clinical pain states and strongly suggests that the current work should be replicated in patient populations to further clarify this issue.

## References

[1] Apkarian AV, Hashmi JA, Baliki MN (2011) Pain and the brain: specificity and plasticity of the brain in clinical chronic pain. Pain 152: S49–64. 21146929 10.1016/j.pain.2010.11.010 PMC3045648

[2] Wand BM, Parkitny L, O’Connell NE, Luomajoki H, McAuley JH, et al (2011) Cortical changes in chronic low back pain: current state of the art and implications for clinical practice. Manual Therapy 16: 15–20. 20655796 10.1016/j.math.2010.06.008

[3] Seifert F, Maihöfner C (2011) Functional and structural imaging of pain-induced neuroplasticity. Current Opinion in Anesthesiology 24: 515–523. 21822136 10.1097/ACO.0b013e32834a1079

[4] Henry DE, Chiodo AE, Yang W (2011) Central nervous system reorganization in a variety of chronic pain states: a review. PM & R 3: 1116–1125. 22192321 10.1016/j.pmrj.2011.05.018

[5] Apkarian AV, Baliki MN, Geha PY (2009) Towards a theory of chronic pain. Progress in Neurobiology 87: 81–97. 18952143 10.1016/j.pneurobio.2008.09.018 PMC2650821

[6] Tracey I, Bushnell MC (2009) How neuroimaging studies have challenged us to rethink: is chronic pain a disease? The Journal Of Pain 10: 1113–1120. 19878862 10.1016/j.jpain.2009.09.001

[7] Wand BM, O'Connell NE (2008) Chronic non-specific low back pain - sub-groups or a single mechanism? BMC Musculoskeletal Disorders 9: 11. 18221521 10.1186/1471-2474-9-11 PMC2266926

[8] Harris AJ (1999) Cortical origin of pathological pain. Lancet 354: 1464–1466. 10543687 10.1016/S0140-6736(99)05003-5

[9] McCabe CS, Blake DR (2007) Evidence for a mismatch between the brain's movement control system and sensory system as an explanation for some pain-related disorders. Current Pain And Headache Reports 11: 104–108. 17367588 10.1007/s11916-007-0006-x

[10] Sperry RW (1950) Neural basis of the spontaneous optokinetic response produced by visual inversion. Journal of Comparative and Physiological Psychology 43: 482–489. 14794830 10.1037/h0055479

[11] Frith CD, Blakemore SJ, Wolpert DM (2000) Abnormalities in the awareness and control of action. Philosophical Transactions Of The Royal Society Of London Series B, Biological Sciences 355: 1771–1788. 11205340 10.1098/rstb.2000.0734 PMC1692910

[12] McCabe CS, Blake DR (2008) An embarrassment of pain perceptions? Towards an understanding of and explanation for the clinical presentation of CRPS type 1. Rheumatology 47: 1612–1616. 18625661 10.1093/rheumatology/ken254

[13] McCabe CS, Haigh RC, Halligan PW, Blake DR (2005) Simulating sensory-motor incongruence in healthy volunteers: implications for a cortical model of pain. Rheumatology 44: 509–516. 15644392 10.1093/rheumatology/keh529

[14] McCabe CS, Cohen H, Blake DR (2007) Somaesthetic disturbances in fibromyalgia are exaggerated by sensory-motor conflict: implications for chronicity of the disease? Rheumatology 46: 1587–1592. 17767000 10.1093/rheumatology/kem204

[15] Daenen L, Nijs J, Roussel N, Wouters K, Van Loo M, et al (2012) Sensorimotor incongruence exacerbates symptoms in patients with chronic whiplash associated disorders: an experimental study. Rheumatology 51: 1492–1499. 22525161 10.1093/rheumatology/kes050

[16] Daenen L, Roussel N, Cras P, Nijs J (2010) Sensorimotor incongruence triggers sensory disturbances in professional violinists: an experimental study. Rheumatology 49: 1281–1289. 20338886 10.1093/rheumatology/keq067

[17] Daenen L, Nijs J, Roussel N, Wouters K, Cras P (2012) Altered perception of distorted visual feedback occurs soon after whiplash injury: an experimental study of central nervous system processing. Pain Physician 15: 405–413. 22996852

[18] Moseley GL, McCormick K, Hudson M, Zalucki N (2006) Disrupted cortical proprioceptive representation evokes symptoms of peculiarity, foreignness and swelling, but not pain. Rheumatology 45: 196–200. 16377731 10.1093/rheumatology/kei119

[19] Fernández-de-las-Peñas C, Pérez-de-Heredia M, Brea-Rivero M, Miangolarra-Page JC (2007) Immediate effects on pressure pain threshold following a single cervical spine manipulation in healthy subjects. Journal of Orthopaedic & Sports Physical Therapy 37: 325–329. 17612359 10.2519/jospt.2007.2542

[20] Chesterton LS, Sim J, Wright CC, Foster NE (2007) Interrater reliability of algometry in measuring pressure pain thresholds in healthy humans, using multiple raters. Clinical Journal of Pain 23: 760–766. 18075402 10.1097/AJP.0b013e318154b6ae

[21] Moseley GL, Gandevia SC (2005) Sensory-motor incongruence and reports of ‘pain’. Rheumatology 44: 1083–1085. 15827043 10.1093/rheumatology/keh631

[22] Foell J, Bekrater-Bodmann R, McCabe CS, Flor H (2013) Sensorimotor incongruence and body perception: An experimental investigation. Frontiers in Human Neuroscience 7: 310. 23805095 10.3389/fnhum.2013.00310 PMC3690352

[23] Mohan R, Jensen KB, Petkova VI, Dey A, Barnsley N, et al (2012) No pain relief with the rubber hand illusion. PLoS One 7: e52400. 23285026 10.1371/journal.pone.0052400 PMC3527497

[24] Moseley GL, Gallace A, Spence C (2008) Is mirror therapy all it is cracked up to be? Current evidence and future directions. Pain 138: 7–10. 18621484 10.1016/j.pain.2008.06.026

[25] Moseley GL, Gallace A, Spence C (2012) Bodily illusions in health and disease: physiological and clinical perspectives and the concept of a cortical ‘body matrix’. Neuroscience And Biobehavioral Reviews 36: 34–46. 21477616 10.1016/j.neubiorev.2011.03.013

[26] Mancini F, Longo MR, Kammers MPM, Haggard P (2011) Visual Distortion of Body Size Modulates Pain Perception. Psychological Science 22: 325–330. 21303990 10.1177/0956797611398496

[27] Moseley GL, Parsons TJ, Spence C (2008) Visual distortion of a limb modulates the pain and swelling evoked by movement. Current Biology 18: R1047–R1048. 19036329 10.1016/j.cub.2008.09.031

[28] Chan BL, Witt R, Charrow AP, Magee A, Howard R, et al (2007) Mirror therapy for phantom limb pain. New England Journal of Medicine 357: 2206–2207. 18032777 10.1056/NEJMc071927

[29] Cacchio A, De Blasis E, De Blasis V, Santilli V, Spacca G (2009) Mirror therapy in complex regional pain syndrome type 1 of the upper limb in stroke patients. Neurorehabilitation & Neural Repair 23: 792–799. 19465507 10.1177/1545968309335977

[30] Moseley GL, Flor H (2012) Targeting Cortical Representations in the Treatment of Chronic Pain: A Review. Neurorehabilitation & Neural Repair 26: 646–652. 22331213 10.1177/1545968311433209

[31] Bowering JK, O’Connell NE, Tabor A, Catley MJ, Leake HB, et al (2013) The effects of graded motor imagery and its components on chronic pain: a systematic review and meta-analysis. *The Journal of Pain* 14: 3–13. 23158879 10.1016/j.jpain.2012.09.007

[32] Wand BM, Tulloch VM, George PJ, Smith AJ, Goucke R, et al (2012) Seeing it helps: movement-related back pain is reduced by visualization of the back during movement. The Clinical Journal Of Pain 28: 602–608. 22699134 10.1097/AJP.0b013e31823d480c

[33] McCabe C (2011) Mirror visual feedback therapy. A practical approach. Journal of hand therapy 24: 170–178. 21106347 10.1016/j.jht.2010.08.003

[34] Kennett S, Taylor-Clarke M, Haggard P (2001) Noninformative vision improves the spatial resolution of touch in humans. Current Biology 11: 1188–1191. 11516950 10.1016/s0960-9822(01)00327-x

[35] Lewis JS, Kersten P, McPherson KM, Taylor GJ, Harris N, et al (2010) Wherever is my arm? Impaired upper limb position accuracy in Complex Regional Pain Syndrome. Pain 149: 463–469. 20385441 10.1016/j.pain.2010.02.007

[36] Ramachandran VS, Altschuler EL (2009) The use of visual feedback, in particular mirror visual feedback, in restoring brain function. Brain 132: 1693–1710. 19506071 10.1093/brain/awp135

[37] Longo MR, Betti V, Aglioti SM, Haggard P (2009) Visually Induced Analgesia: Seeing the Body Reduces Pain. Journal of Neuroscience 29: 12125–12130. 19793970 10.1523/JNEUROSCI.3072-09.2009 PMC6666129

[38] Moseley GL (2007) Reconceptualising pain according to modern pain science. Physical Therapy Reviews 12: 169–178.

